# SPECIFIC: A systematic framework for engineering cell state‐responsive synthetic promoters reveals key regulators of T cell exhaustion

**DOI:** 10.1002/qub2.97

**Published:** 2025-02-20

**Authors:** Zhaoyu Zhang, Xiaoyu Qiu, Hui Ning, Zihua Huang, Minzhen Tao, Min Liang, Zhen Xie

**Affiliations:** ^1^ MOE Key Laboratory of Bioinformatics and Bioinformatics Division Center for Synthetic and Systems Biology Department of Automation Beijing National Research Center for Information Science and Technology Tsinghua University Beijing China; ^2^ Hesheng Beiyin Inc. Qingdao China

**Keywords:** promoter, synthetic biology, synthetic promoter, T cell exhaustion

## Abstract

Cell state‐specific synthetic promoters are essential tools for studying and manipulating cellular function, yet their design remains challenging, particularly for complex states such as T cell exhaustion. Here we present SPECIFIC (Synthetic Promoter Engineering for Cellular State Identification and Functional Analysis), an integrated framework that leverages chromatin accessibility profiling and machine learning to systematically identify and validate cell state‐specific synthetic promoters. By comparing exhausted T cells from both mouse OT‐I and human CAR‐T models, we identified 56 conserved transcription factor binding motifs associated with T cell exhaustion. From these motifs, we engineered a subset of the most promising candidates into synthetic promoters driving an exhaustion‐responsive gene circuit that senses and responds to T cell dysfunction. Several synthetic promoters, particularly those containing NFATc2 or MEF2C binding sites, demonstrated remarkable specificity in recognizing the exhausted state and effectively attenuated T cell dysfunction by reducing both CAR expression and exhaustion markers. This study establishes a generalizable approach for designing cell state‐specific regulatory elements and provides new strategies for improving CAR‐T cell therapy through programmed control of gene expression.

## 1 INTRODUCTION

The ability to precisely control gene expression in response to specific cellular states represents a fundamental goal in synthetic biology and therapeutic development. Synthetic promoters with enhanced cell‐state specificity offer powerful tools for both mechanistic studies [1, 2] and therapeutic applications [3, 4, 5] as they can activate gene expression selectively under defined biological conditions. However, designing such promoters remains challenging, particularly for complex cellular states that involve multiple regulatory networks and dynamic transitions. This challenge is exemplified in the context of T cell exhaustion [6], a dysfunctional state that significantly limits immunotherapy efficacy.

T cell exhaustion, characterized by progressive loss of effector functions and proliferative capacity, emerges as a critical barrier in cancer immunotherapy and chronic infection control. This dysfunctional state is marked by elevated expression of multiple inhibitory receptors, impaired cytokine production, and distinct transcriptional programs. Recent studies have revealed key transcription factors, including NFAT [7], IRF4 [8], c‐Jun [9], TOX [10, 11], and NR4A1 [12], that orchestrate exhaustion‐specific gene expression through complex epigenetic modifications. Despite these advances, the precise regulatory networks governing the transition from functional to exhausted T cells remain incompletely understood, largely due to the lack of tools that can accurately monitor and modulate state‐specific gene expression.

Traditional approaches to synthetic promoter design often rely on empirical testing of limited transcription factor binding sites or based on the prior knowledge of gene regulatory pathways [5, 13]. Although these methods have yielded valuable insights, they frequently result in promoters with suboptimal specificity and limited functionality. Recent high‐throughput screening approaches such as SPECS (Synthetic Promoters with Enhanced Cell‐State Specificity) [14] enable systematic testing of synthetic promoters, but the extensive experimental requirements and lack of systematic approaches for prioritizing candidate transcription factor binding sites make this approach challenging to implement effectively. The emergence of high‐throughput genomic technologies, particularly ATAC‐seq [15] and single‐cell transcriptomics [16], offers unprecedented opportunities to comprehensively map the regulatory landscapes of distinct cellular states. However, effectively translating this wealth of genomic data into rational design principles for synthetic promoters remains a significant challenge.

To address these limitations, we developed SPECIFIC (Synthetic Promoter Engineering for Cellular State Identification and Functional Analysis), a systematic framework that integrates chromatin accessibility profiling, computational motif analysis, and synthetic biology approaches. This pipeline combines computational identification of state‐specific regulatory elements with rational design principles to generate synthetic promoters with enhanced cellular state specificity. By leveraging cross‐species comparative analysis between mouse and human T cell exhaustion models, we aimed to identify conserved regulatory elements that could serve as building blocks for exhaustion‐specific synthetic promoters.

Through this integrated approach, we identified a comprehensive set of transcription factor binding motifs specifically enriched in exhausted T cells and engineered them into synthetic promoters capable of sensing the exhausted state. These promoters were incorporated into gene circuits designed to counteract T cell exhaustion, demonstrating both the analytical and therapeutic potential of our approach. The SPECIFIC pipeline not only advances our understanding of T cell exhaustion but also provides a generalizable framework for engineering synthetic promoters responsive to any well‐defined cellular state. This work establishes a new paradigm for rational design of cell state‐specific regulatory elements and offers innovative strategies for improving cellular therapies through programmed gene regulation.

## 2 RESULTS

### 2.1 SPECIFIC pipeline for cell state‐specific synthetic promoter design

**FIGURE 1:**
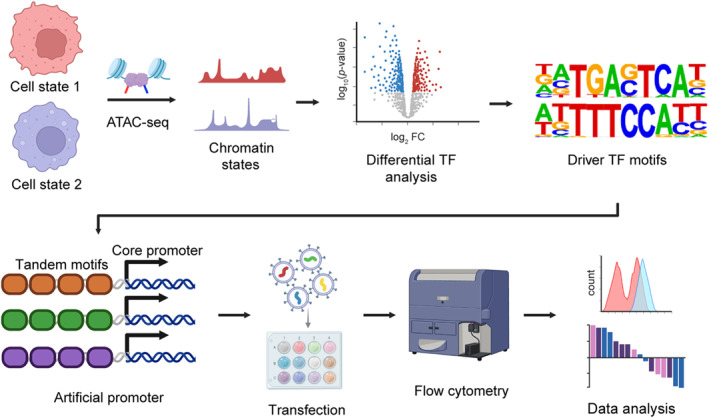
The SPECIFIC pipeline for systematic identification of cell state‐specific synthetic promoters. Schematic overview of the SPECIFIC workflow. Cell state comparison begins with ATAC‐seq profiling of two distinct cellular states to map genome‐wide chromatin accessibility. Differential analysis of chromatin states identifies state‐specific accessible regions, followed by computational identification of enriched TF binding motifs. These driver TF motifs are then engineered into synthetic promoters by arranging them in tandem arrays upstream of a core promoter. The resulting synthetic promoters are tested through lentiviral delivery and flow cytometry analysis to validate their state specificity and regulatory activity. Schematic illustration figures were created with BioRender.com with publication licenses. Source data are provided as a Source Data file. TF, transcription factor.

We established SPECIFIC (Synthetic Promoter Engineering for Functional Identification of Cellular States), a comprehensive framework that integrates chromatin accessibility profiling, computational motif analysis, and synthetic biology approaches to generate cell state‐specific synthetic promoters (Figure 1). This pipeline systematically identifies and validates regulatory elements that drive distinct cellular states through sequential analytical and experimental phases.

The SPECIFIC workflow initiates with ATAC‐seq analysis of cells in distinct physiological or pathological states to delineate genome‐wide chromatin accessibility landscapes. The accessibility profiles undergo computational analysis using Homer [17] to identify differentially enriched transcription factor (TF) binding motifs between distinct cellular states. This approach enables the identification of putative regulatory elements that potentially orchestrate state‐specific gene expression programs.

Leveraging design principles from synthetic promoter engineering, we constructed artificial promoters by arranging tandem arrays of the identified state‐specific TF binding motifs upstream of a minimal CMV promoter. This design strategy, inspired by previous work on synthetic promoters with enhanced cell‐state specificity (SPECS) [14], creates combinatorial regulatory elements capable of sensing and responding to cellular state‐specific transcription factor activities.

To validate the functionality of these synthetic promoters, we employed a systematic screening approach. The designed promoter sequences were synthesized and cloned into plasmid or lentiviral vectors for cellular delivery. The activity and specificity of each synthetic promoter were then quantitatively assessed using flow cytometry, enabling precise measurement of reporter gene expression across different cellular states. This experimental validation pipeline facilitates the identification of synthetic promoters that exhibit robust state‐specific activity.

Through this integrated approach, SPECIFIC provides a generalizable framework for engineering synthetic promoters with enhanced cellular state specificity, offering a valuable tool for studying and manipulating cell state transitions in various biological contexts.

### 2.2 Identification of T cell state‐specific TF motifs

To systematically identify transcription factor motifs associated with T cell exhaustion, we integrated ATAC‐seq data from three independent exhaustion models. First, we utilized an in vitro mouse exhaustion model [18] based on repetitive antigen stimulation of OT‐I mouse T cells with OVA peptide. This model rapidly induces hallmark features of T cell exhaustion within 5 days of continuous stimulation, including decreased cytokine production, elevated inhibitory receptor expression, and impaired proliferative capacity. We performed ATAC‐seq on OT‐I mouse T cells after 1 day (nonexhausted, cytotoxic state) and 5 days (exhausted state) of stimulation to capture the chromatin accessibility landscapes associated with these distinct functional states. Second, we analyzed published ATAC‐seq data from tumor‐infiltrating exhausted OT‐I T cells in a mouse melanoma model [7]. Additionally, we leveraged the GD2‐targeting HA‐28z CAR T cell model [19], which exhibits pronounced exhaustion during ex vivo expansion compared to CD19‐targeting CAR T cells. This system provides a human‐relevant context for studying exhaustion‐associated chromatin dynamics. We analyzed published ATAC‐seq datasets from HA‐28z CAR T cells (exhausted) and CD19 CAR T cells (nonexhausted) to delineate exhaustion‐specific regulatory elements in human T cells.

**FIGURE 2:**
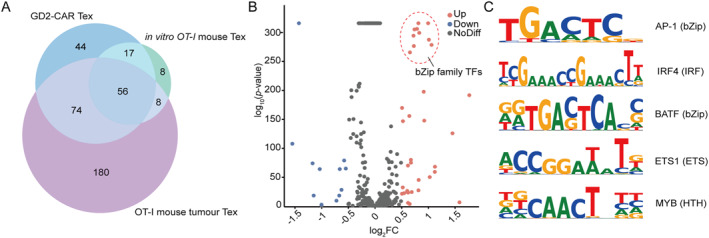
Identification of conserved transcription factor motifs associated with T cell exhaustion. (A) Venn diagram showing the overlap of differentially enriched transcription factor binding motifs among 3 T cell exhaustion models: in vitro exhausted OT‐I mouse T cells, tumor‐infiltrating exhausted OT‐I mouse T cells, and exhausted GD2‐CAR T cells. 56 transcription factor binding motifs were found to be enriched across all three models. (B) Volcano plot showing differential chromatin accessibility analysis of exhausted versus nonexhausted T cells. Red dots indicate regions with increased accessibility in exhausted cells, with bZIP family transcription factors notably enriched (circled). Blue dots indicate decreased accessibility, and gray dots represent regions with no significant change. (C) Sequence logos of representative transcription factor binding motifs from different families enriched in exhausted T cells, including AP‐1 (bZIP), IRF4 (IRF), BATF (bZIP), ETS1 (ETS), and MYB (HTH).

To identify transcription factor motifs enriched in exhausted T cells, we performed differential motif analysis using Homer [17] on all three ATAC‐seq datasets. By intersecting motifs that showed significant enrichment (*p* < 0.05) across all three datasets, we identified 56 transcription factor binding motifs consistently associated with the exhausted state (Figure 2A). The differential motif analysis across all three datasets revealed consistent enrichment of distinct transcription factor families, particularly the bZIP family members. This enrichment is exemplified in the volcano plot analysis of the GD2 CAR dataset, where bZIP transcription factors such as JunB, Fosl2, and BATF formed a prominent cluster of highly enriched regulators (Figure 2B). Among the significantly enriched motifs in exhausted T cells, we identified several key transcription factors, including representatives from multiple families known to regulate T cell exhaustion: bZIP family members (AP‐1, BATF), IRF family members (IRF4), ETS family members (ETS1), and HTH family members (MYB). The sequence logos of these representative motifs highlight their distinct binding preferences (Figure 2C).

This cross‐species comparative analysis provides a comprehensive map of regulatory elements potentially driving T cell exhaustion, thereby establishing a robust foundation for the rational design of exhaustion‐specific synthetic promoters. The conservation of these motifs between mouse and human exhaustion models suggests their fundamental importance in orchestrating the exhaustion program.

### 2.3 Construction of synthetic promoters for exhaustion state recognition

**FIGURE 3:**
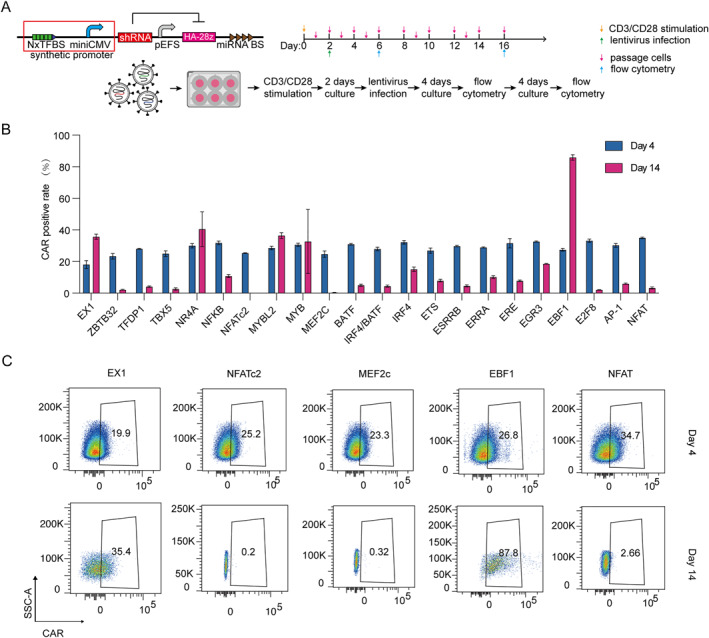
Design and validation of exhaustion‐responsive synthetic promoters. (A) Schematic of the gene circuit design. The synthetic promoter containing tandem transcription factor binding sites drives expression of shRNA targeting CAR transcripts. The complete cassette includes a miniCMV promoter, shRNA, and miRNA binding sites. Timeline shows the experimental workflow for testing synthetic promoters in primary T cells. (B) Quantification of CAR expression in T cells containing different synthetic promoters at day 4 (blue) and day 14 (pink) post‐infection. EX1 serves as a control construct lacking TF binding sites. Data shown as mean ± SEM (*n* = 3). (C) Representative flow cytometry plots showing CAR expression levels in T cells containing indicated synthetic promoters at day 4 and day 14 post‐infection.

Based on our cross‐species comparative transcription factor analysis, we designed synthetic state‐specific promoters incorporating binding sites from three key sources: transcription factors showing consistent enrichment across our mouse and human exhaustion models, factors demonstrating significant enrichment in individual models, and established exhaustion‐associated factors such as TOX [10, 20] from previous studies. In total, we selected 22 transcription factors to engineer into synthetic promoters, with the classical NFAT promoter [21] serving as a control based on its well‐characterized role in T cell regulation. To test these promoters’ ability to regulate T cell exhaustion, we engineered a gene circuit in which the synthetic promoter drives expression of a microRNA (FF4) targeting GD2‐CAR transcripts (Figure 3A). Specifically, tandem arrays of transcription factor binding sites were positioned upstream of a minimal CMV promoter controlling FF4 expression, whereas GD2‐CAR was expressed from an EFS promoter with microRNA binding sites in its 3’UTR. In this design, exhaustion‐associated transcription factors would activate the synthetic promoter, leading to FF4‐mediated suppression of CAR expression and thereby potentially attenuating exhaustion driven by tonic CAR signaling.

After introducing these gene circuits into primary human T cells via lentiviral vectors, we monitored CAR expression over 14 days of culture. Initial analysis at day 4 post‐infection showed comparable CAR expression levels across all constructs, with positivity rates ranging from 25% to 40%. The control construct (EX1), lacking any transcription factor binding sites, maintained stable CAR expression (about 35% CAR^+^ cells) through day 14, confirming that the basic circuit architecture does not intrinsically affect CAR expression. In contrast, several synthetic promoters demonstrated remarkable state‐specificity over time. Most notably, constructs containing NFATc2 or MEF2c binding sites showed nearly complete suppression of CAR expression by day 14 (Figure 3B,C), suggesting these transcription factors are particularly active in the exhausted state and capable of robustly activating their cognate synthetic promoters.

**FIGURE 4:**
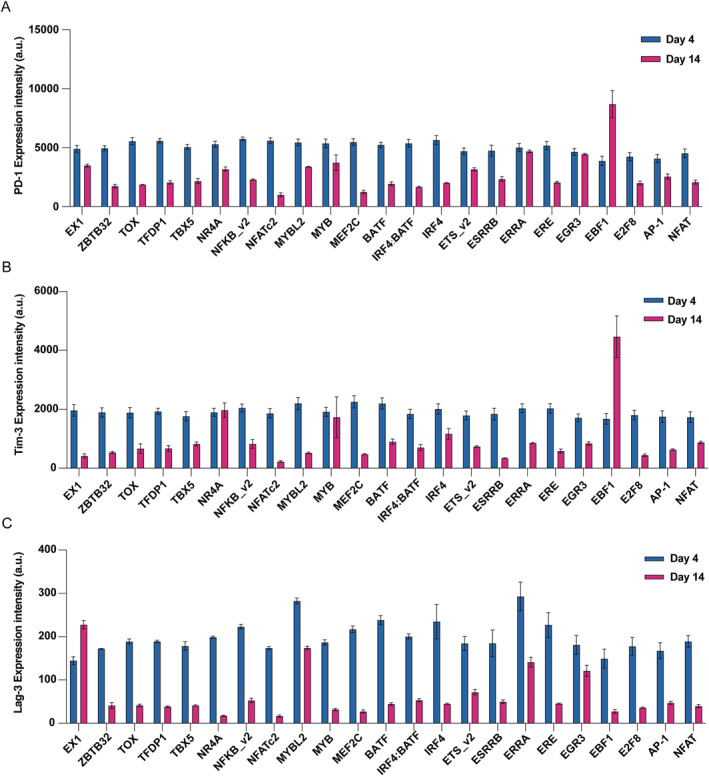
NFATc2 and MEF2C promoter‐driven circuits effectively attenuate T cell exhaustion markers. Expression of exhaustion markers in T cells containing different synthetic promoters at day 4 (blue) and day 14 (pink) post‐infection. (A) PD‐1 expression levels. (B) Tim‐3 expression levels. (C) LAG‐3 expression levels. NFATc2 and MEF2C promoter‐containing circuits show coordinated reduction in both CAR expression and exhaustion markers, whereas other constructs (e.g., ERRA and EGR3) show varied effects. Data shown as mean ± SEM (*n* = 3). Expression intensity measured in arbitrary units (a.u.).

We further examined whether reduced CAR expression correlated with attenuation of the exhaustion phenotype by measuring key exhaustion markers. Strikingly, the NFATc2 and MEF2c promoter‐driven circuits not only suppressed CAR expression but also maintained significantly lower levels of PD‐1, TIM‐3, and LAG‐3 at day 14 compared to the control construct (Figure 4A–C). This coordinated reduction in both CAR and exhaustion markers suggests successful interruption of the feed‐forward loop between tonic CAR signaling and exhaustion programming. Interestingly, some constructs showed divergent effects on different markers. For example, circuits incorporating ERRA or EGR3 binding sites achieved substantial CAR suppression but showed minimal impact on PD‐1 and LAG‐3 levels, whereas TIM‐3 expression appeared less consistently regulated across all constructs. These variations might reflect the involvement of different transcription factors in distinct aspects or temporal phases of the exhaustion program. Together, these results validate our SPECIFIC pipeline’s ability to identify the true state‐specific synthetic promoters and demonstrate the feasibility of using exhaustion‐responsive gene circuits to counteract T cell dysfunction. This approach provides both valuable tools for studying exhaustion mechanisms and potential strategies for improving CAR T cell therapy through programmed regulation of CAR expression.

## 3 DISCUSSION

Our study demonstrates that systematic analysis of chromatin accessibility landscapes can guide the rational design of synthetic promoters with enhanced cell‐state specificity. By leveraging cross‐species comparative analysis between mouse and human T cell exhaustion models, we identified conserved transcriptional regulatory elements that effectively sense and respond to T cell dysfunction. The success of this approach not only validates the SPECIFIC pipeline but also provides new insights into the regulatory mechanisms governing T cell exhaustion.

The identification of NFATc2 binding sites as particularly effective elements for exhaustion‐specific promoters aligns with and extends previous studies of T cell regulation [7, 22]. Intriguingly, our results also revealed MEF2C as a potential potent regulator of the exhausted state—a finding that expands our understanding of this transcription factor beyond its known roles in muscle, neural, and immune cell development [23]. Although MEF2C has been implicated in various aspects of immune cell biology, its specific contribution to T cell exhaustion remains unexplored and warrants further investigation. The remarkable specificity achieved by promoters containing these binding sites suggests that they may serve as core components in the exhaustion‐specific transcriptional network. It is worth noting that the differential effects observed with various transcription factor binding sites—from highly effective (NFATc2 and MEF2C) to intermediate (ERRA and EGR3)—provide new insights into the hierarchical organization of exhaustion‐associated regulatory factors.

Our gene circuit design, which couples exhaustion‐sensing promoters to CAR expression control, represents a novel approach to managing T cell dysfunction. By enabling programmed reduction of CAR expression in response to exhaustion‐associated transcription factors, this system creates a negative feedback loop that may help prevent or delay the onset of exhaustion. The coordinated decrease in both CAR expression and exhaustion markers (PD‐1, TIM‐3 and LAG‐3) in cells containing NFATc2‐ or MEF2C‐driven circuits suggests that modulating CAR levels could be an effective strategy for maintaining T cell functionality. This finding has important implications for improving CAR‐T cell therapy, as it suggests that fine‐tuning receptor expression levels based on cell state could enhance therapeutic efficacy.

The SPECIFIC pipeline offers several advantages over traditional approaches to synthetic promoter design [13]. First, by integrating chromatin accessibility data from multiple models, it enables the identification of truly conserved regulatory elements, potentially reducing species‐specific effects. Second, the systematic screening of a large panel of transcription factor binding sites provides comprehensive coverage of potential regulatory elements, thereby mitigating bias in promoter design. Third, the use of a gene circuit architecture allows for functional validation of promoter specificity through direct measurement of both input (exhaustion markers) and output (CAR expression) parameters.

However, several limitations and areas for future investigation should be noted. Although our current gene circuit design effectively reduces CAR expression in exhausted cells, alternative circuit architectures might provide even better control over T cell function. Furthermore, it is important to acknowledge that both NFATc2 and MEF2C are known to participate in a variety of cellular processes beyond T cell exhaustion, raising the possibility of unintended off‐target effects when using these elements in synthetic promoters. To minimize such risks, we propose several strategies for future development of this technology. These include: (1) employing combinatorial approaches that necessitate the activity of multiple transcription factors for promoter activation, thereby enhancing cell‐state specificity; (2) implementing precise temporal control mechanisms, such as inducible systems, to limit promoter activity to the desired time window; and (3) conducting thorough validation studies in a variety of relevant cellular contexts to assess promoter specificity across different cell types and states. Future studies will focus on investigating the specificity of these promoters in diverse cellular contexts and optimizing their design to minimize off‐target activity. Additionally, although this study focuses on T cell exhaustion, the modular design and computational framework of SPECIFIC are adaptable to other cellular contexts, representing a crucial area for future research. Finally, the long‐term stability and safety of these synthetic promoters in therapeutic applications remain to be fully evaluated. Future studies should also explore whether combining multiple binding sites or incorporating additional regulatory elements could further enhance promoter specificity and robustness.

In summary, the SPECIFIC framework represents a significant advancement in the rational design of cell state‐specific synthetic promoters. Its successful application to T cell exhaustion not only provides a valuable tool for studying this complex state but also offers a promising avenue for improving CAR‐T cell therapy. The principles and methodologies established in this study are expected to be broadly applicable to other cellular states and therapeutic contexts, paving the way for more precise and effective control of gene expression in diverse biological systems.

## 4 CONCLUSION

In conclusion, our work establishes a powerful and broadly applicable approach, termed SPECIFIC, for engineering synthetic promoters with enhanced cell‐state specificity. We demonstrate its utility by addressing a major challenge in immunotherapy: T cell exhaustion. The successful application of SPECIFIC in the context of T cell exhaustion, validated by the identification of key regulatory elements such as NFATc2 and MEF2C, suggests broad potential applications in synthetic biology and therapeutic development. By enabling precise control of gene expression based on cellular state, this technology could significantly advance our ability to develop more effective cell‐based therapies. Future work will focus on expanding the application of SPECIFIC to diverse cell types and states, refining promoter design to minimize off‐target effects, and rigorously evaluating the long‐term safety and efficacy of these synthetic promoters in therapeutic settings. The SPECIFIC framework represents a significant step toward a future where cellular therapies can be dynamically tailored to the evolving state of the target cells, ultimately leading to more effective and personalized treatments.

## 5 MATERIALS AND METHODS

### 5.1 Rapid in vitro generation of mouse exhausted CD8^+^ T cells

Following the protocol established by Zhao et al. [18], murine CD8^+^ T cells were isolated from OT‐I mouse spleens via positive magnetic selection using CD8a (Ly‐2) MicroBeads (Miltenyi Biotec). Isolated cells were cultured at 1 × 10^6^ cells/mL in complete RPMI 1640 medium supplemented with 10% FBS, 2 mmol/L L‐glutamine, 100 nmol/L sodium pyruvate, nonessential amino acids, 100 U/mL penicillin, 100 μg/mL streptomycin, and 0.05 mmol/L β‐mercaptoethanol. Cultures were maintained with IL‐15 (5 ng/mL) and IL‐7 (5 ng/mL) (both from Peprotech). For exhaustion induction, cells were stimulated with 10 ng/mL OVA_257–264_ peptide (Solarbio) daily for 5 days. Control nonexhausted cells received single peptide stimulation for 48 h followed by culture without peptide. Cell density was monitored daily and maintained at approximately 1 × 10^6^ cells/mL through splitting when necessary.

### 5.2 ATAC‐seq NGS sequencing

ATAC‐seq was performed on 100,000 viable cells following established protocols [24]. Briefly, cells were lysed in ATAC‐seq resuspension buffer containing 0.1% NP‐40, 0.1% Tween‐20%, and 0.01% digitonin. Nuclei were isolated and subjected to tagmentation using Tn5 transposase (Vazyme) in transposition buffer for 30 min at 37°C. Tagmented DNA was purified using DNA Clean & Concentrator‐5 (Zymo) and amplified with custom‐indexed primers using NEBNext High‐Fidelity PCR Master Mix (NEB). Libraries were size‐selected for 300–500bp fragments and sequenced on Illumina NextSeq with paired‐end 150bp reads.

### 5.3 Differential transcription factor motif analysis

ATAC‐seq was performed on our OT‐I mouse T cells at day 1 (nonexhausted) and day 5 (exhausted) post‐stimulation. For comparative analysis, we integrated published ATAC‐seq data from tumor‐infiltrating exhausted OT‐I T cells [7] and human GD2‐targeting HA‐28z (exhausted) versus CD19‐targeting (nonexhausted) CAR T cells [19]. ATAC‐seq reads were aligned to mouse genome (mm9) or human genome (hg19) using Bowtie2 with parameters optimized for transposase‐accessible chromatin analysis. For differential motif analysis, Homer [17] was employed to identify enriched transcription factor binding motifs in differential peaks between exhausted and nonexhausted states (fold change > 2, FDR < 0.05). Common motifs between mouse and human datasets were identified by intersecting significantly enriched motifs (*p* < 0.05) from both analyses.

### 5.4 Synthetic promoter design

Based on our differential motif analysis results, we selected 22 transcription factors significantly enriched in both mouse and human exhausted T cells for synthetic promoter design. Following the approaches described by SPECS [14], position weight matrices (PWMs) for selected transcription factors were primarily obtained from the JASPAR database, with some motifs sourced from Homer‐generated PWMs during differential analysis. The TOX binding motif was obtained from previous article [20]. Synthetic promoters were constructed by arranging tandem repeats of identified transcription factor binding sites (Table S1) upstream of a minimal CMV promoter, with 3bp spacers between repeats. Classical NFAT promoter served as a control, while NR4A promoter design followed the prepublished [3] architecture. The number of repeats was calculated to achieve approximately 120bp of binding sites. The oligonucleotides containing tandem TF binding sites were synthesized (Tsingke Biotech) and cloned into lentiviral vectors upstream of a miniCMV promoter controlling shRNA‐FF4 expression (Table S2).

### 5.5 Cloning of lentiviral vectors

The lentiviral backbone was engineered to contain the restriction enzyme cloning sites, mini‐CMV promoter, shRNA‐FF4, and EFS promoter driving the expression of HA‐28z GD2‐targeting CAR gene (Table S2). Synthetic TF motifs were cloned into the backbone using conventional restriction enzyme cloning. Plasmid quality was verified by Sanger sequencing.

### 5.6 Lentivirus production

HEK293T cells were co‐transfected with packaging plasmids (1 μg VSV.G) envelope plasmid, 2 μg psPAX2, and 4 μg synthetic promoter transfer plasmid using EpFect transfection reagent (SyngenTech). Culture medium was replaced 18 h post‐transfection and virus‐containing supernatant was collected at 48 h. Viral particles were concentrated by filtration through 0.45 μm filters and stored at −80°C until use.

### 5.7 Primary human T cell stimulation and culture

Primary human T cells were purchased from Milestone Biological Science & Technology Co., Ltd. Cells were maintained in complete RPMI 1640 medium supplemented with 10% FBS, 55 μmol/L β‐Mercaptoethanol, 300 U/mL IL‐2, and 100 mmol/L L‐glutamine. For activation, cells were stimulated with anti‐CD3/CD28 nanomagnetic bead (Miltenyi Biotec). Cell density was maintained between 0.5 × 10^6^ cells/mL and 2 × 10^6^ cells/mL.

### 5.8 Lentiviral transduction of primary human T cells

After 48 h stimulation, the nanobeads were removed and stimulated T cells were transduced with lentiviral particles at MOI 10 for 2 days. Transduced cells were expanded for 5–14 days before analysis.

### 5.9 Flow cytometry

Cell surface markers were analyzed by flow cytometry using fluorochrome‐conjugated antibodies. Transduced T cells were analyzed on days 4 and 14 post‐lentiviral infection for CAR expression and exhaustion markers including PD‐1, TIM‐3, and LAG‐3. For surface staining, cells were incubated with antibodies in FACS buffer (PBS with 2% BSA) for 30 min at 4°C. Flow cytometry data were acquired on a BD LSRFortessa analyzer and analyzed using FlowJo software (TreeStar).

## AUTHOR CONTRIBUTIONS

**Zhaoyu Zhang:** Conceptualization; formal analysis; investigation; methodology; project administration; software; visualization; writing—original draft; writing—review and editing. **Xiaoyu Qiu:** Data curation; formal analysis; investigation; methodology; validation; visualization; writing—review and editing. **Hui Ning:** Conceptualization; investigation; methodology; validation; writing—review and editing. **Zihua Huang:** Data curation; investigation; software. **Minzhen Tao:** Writing—review and editing. **Min Liang:** Resources. **Zhen Xie:** Conceptualization; funding acquisition; project administration; resources; supervision; writing—review and editing.

## CONFLICT OF INTEREST STATEMENT

Zhen Xie, Zhaoyu Zhang, Zihua Huang, and Shuo Zhang have registered a computer software copyright based on the presented work with the National Copyright Administration of China (Registration No. 2024SR1789163). Tsinghua University is the owner of the copyright. The remaining authors except Zhen Xie, who is the Assistant Editor‐in‐Chief, declare no competing interests. Zhen Xie was excluded from the peer‐review process and all editorial decisions related to the acceptance and publication of this article. Peer review was handled independently by the other editors to minimize bias.

## ETHICS STATEMENT

No additional ethics statement should be disclosed in this study.

## Supporting information

Supporting Information S1

## Data Availability

The transcription factors and their corresponding synthetic promoter sequences used in this study are provided in the Supplementary Tables.
