# Supplementary material for: SPECIFIC: A systematic framework for engineering cell state‐responsive synthetic promoters reveals key regulators of T cell exhaustion
Source: Quant Biol. 2025 Feb 20;13(2):e97. doi: 10.1002/qub2.97 (PMC12806122; doi:10.1002/qub2.97)
Supplement: Supplementary file 1 — Supporting Information S1 [file QUB2-13-e97-s001.docx]

**Table S1.** Transcription factors’ name and sequence

| TF name | Motif sequence | Promoter Sequence (without miniCMV) |
| --- | --- | --- |
| AP-1 | TTGAGTCA | TTGAGTCAAGATTGAGTCATCGTTGAGTCAGACTTGAGTCACTATTGAGTCAACTTTGAGTCATGCTTGAGTCAGTATTGAGTCA |
| BATF (bZIP family: JunD, Fra1, Fra2, ATF...） | GGTGACTCAT | GGTGACTCATAGAGGTGACTCATTCGGGTGACTCATGACGGTGACTCATCTAGGTGACTCATACTGGTGACTCATTGCGGTGACTCATGTAGGTGACTCAT |
| E2F8 | TTTTGGCGGGAAAA | TTTTGGCGGGAAAAAGATTTTGGCGGGAAAATCGTTTTGGCGGGAAAAGACTTTTGGCGGGAAAACTATTTTGGCGGGAAAAACTTTTTGGCGGGAAAATGCTTTTGGCGGGAAAAGTATTTTGGCGGGAAAA |
| EBF1 | GTCCCCAGGGGA | GTCCCCAGGGGAAGAGTCCCCAGGGGATCGGTCCCCAGGGGAGACGTCCCCAGGGGACTAGTCCCCAGGGGAACTGTCCCCAGGGGA |
| EGR3 | ACGCCCACGCA | ACGCCCACGCAAGAACGCCCACGCATCGACGCCCACGCAGACACGCCCACGCACTAACGCCCACGCAACTACGCCCACGCATGCACGCCCACGCAGTAACGCCCACGCA |
| ERE | AAGGTCACTCTGACC | AAGGTCACTCTGACCAGAAAGGTCACTCTGACCTCGAAGGTCACTCTGACCGACAAGGTCACTCTGACCCTAAAGGTCACTCTGACCACTAAGGTCACTCTGACC |
| ERRA | CAAAGGTCAG | CAAAGGTCAGAGACAAAGGTCAGTCGCAAAGGTCAGGACCAAAGGTCAGCTACAAAGGTCAGACTCAAAGGTCAGTGCCAAAGGTCAGGTACAAAGGTCAG |
| ESRRB | GTGACCTTGA | GTGACCTTGAAGAGTGACCTTGATCGGTGACCTTGAGACGTGACCTTGACTAGTGACCTTGAACTGTGACCTTGATGCGTGACCTTGAGTAGTGACCTTGA |
| ETS | CCGGAAGTGGC | CCGGAAGTGGCAGACCGGAAGTGGCTCGCCGGAAGTGGCGACCCGGAAGTGGCCTACCGGAAGTGGCACTCCGGAAGTGGCTGCCCGGAAGTGGCGTACCGGAAGTGGC |
| IRF4 | CCGAAACCGAAACTA | CCGAAACCGAAACTAAGACCGAAACCGAAACTATCGCCGAAACCGAAACTAGACCCGAAACCGAAACTACTACCGAAACCGAAACTAACTCCGAAACCGAAACTA |
| BATF/IRF4 | GAAATGAGTCA | GAAATGAGTCAAGAGAAATGAGTCATCGGAAATGAGTCAGACGAAATGAGTCACTAGAAATGAGTCAACTGAAATGAGTCATGCGAAATGAGTCAGTAGAAATGAGTCA |
| MEF2c | ACCAAAAATAGA | ACCAAAAATAGAAGAACCAAAAATAGATCGACCAAAAATAGAGACACCAAAAATAGACTAACCAAAAATAGAACTACCAAAAATAGA |
| MYB | CTCAACTGGC | CTCAACTGGCAGACTCAACTGGCTCGCTCAACTGGCGACCTCAACTGGCCTACTCAACTGGCACTCTCAACTGGCTGCCTCAACTGGCGTACTCAACTGGC |
| MYBL2 | AACCGTTAAACGGTC | AACCGTTAAACGGTCAGAAACCGTTAAACGGTCTCGAACCGTTAAACGGTCGACAACCGTTAAACGGTCCTAAACCGTTAAACGGTCACTAACCGTTAAACGGTC |
| NFATc2 | ATGGAAAA | ATGGAAAAAGAATGGAAAATCGATGGAAAAGACATGGAAAACTAATGGAAAAACTATGGAAAATGCATGGAAAAGTAATGGAAAA |
| NFKB | AGGGGATTTCCAAGG | AGGGGATTTCCAAGGAGAAGGGGATTTCCAAGGTCGAGGGGATTTCCAAGGGACAGGGGATTTCCAAGGCTAAGGGGATTTCCAAGGACTAGGGGATTTCCAAGG |
| NR4A | AAAGGTCAC | AAAGGTCACAGAAAAGGTCACTCGAAAGGTCACGACAAAGGTCACCTAAAAGGTCACACTAAAGGTCACTGCAAAGGTCACGTAAAAGGTCAC |
| TBX5 | AGGTGTCA | AGGTGTCAAGAAGGTGTCATCGAGGTGTCAGACAGGTGTCACTAAGGTGTCAACTAGGTGTCATGCAGGTGTCAGTAAGGTGTCA |
| TFDP1 | GTTTCCCGCCATTT | GTTTCCCGCCATTTAGAGTTTCCCGCCATTTTCGGTTTCCCGCCATTTGACGTTTCCCGCCATTTCTAGTTTCCCGCCATTTACTGTTTCCCGCCATTT |
| TOX | GGCGGGGGCG | GGCGGGGGCGAGAGGCGGGGGCGTCGGGCGGGGGCGGACGGCGGGGGCGCTAGGCGGGGGCGACTGGCGGGGGCGTGCGGCGGGGGCGGTAGGCGGGGGCG |
| ZBTB32 | TGTACAGTATTACTGTACA | TGTACAGTATTACTGTACAAGATGTACAGTATTACTGTACATCGTGTACAGTATTACTGTACAGACTGTACAGTATTACTGTACACTATGTACAGTATTACTGTACA |
| ESRRB | GTGACCTTGA | GTGACCTTGAAGAGTGACCTTGATCGGTGACCTTGAGACGTGACCTTGACTAGTGACCTTGAACTGTGACCTTGATGCGTGACCTTGAGTAGTGACCTTGA |
| ETS | CCGGAAGTGGC | CCGGAAGTGGCAGACCGGAAGTGGCTCGCCGGAAGTGGCGACCCGGAAGTGGCCTACCGGAAGTGGCACTCCGGAAGTGGCTGCCCGGAAGTGGCGTACCGGAAGTGGC |
| IRF4 | CCGAAACCGAAACTA | CCGAAACCGAAACTAAGACCGAAACCGAAACTATCGCCGAAACCGAAACTAGACCCGAAACCGAAACTACTACCGAAACCGAAACTAACTCCGAAACCGAAACTA |
| BATF/IRF4 | GAAATGAGTCA | GAAATGAGTCAAGAGAAATGAGTCATCGGAAATGAGTCAGACGAAATGAGTCACTAGAAATGAGTCAACTGAAATGAGTCATGCGAAATGAGTCAGTAGAAATGAGTCA |
| MEF2c | ACCAAAAATAGA | ACCAAAAATAGAAGAACCAAAAATAGATCGACCAAAAATAGAGACACCAAAAATAGACTAACCAAAAATAGAACTACCAAAAATAGA |
| MYB | CTCAACTGGC | CTCAACTGGCAGACTCAACTGGCTCGCTCAACTGGCGACCTCAACTGGCCTACTCAACTGGCACTCTCAACTGGCTGCCTCAACTGGCGTACTCAACTGGC |
| MYBL2 | AACCGTTAAACGGTC | AACCGTTAAACGGTCAGAAACCGTTAAACGGTCTCGAACCGTTAAACGGTCGACAACCGTTAAACGGTCCTAAACCGTTAAACGGTCACTAACCGTTAAACGGTC |
| NFATc2 | ATGGAAAA | ATGGAAAAAGAATGGAAAATCGATGGAAAAGACATGGAAAACTAATGGAAAAACTATGGAAAATGCATGGAAAAGTAATGGAAAA |

**Table S2.** Genetic parts sequences

| Parts name | Sequence |
| --- | --- |
| miniCMV promoter | GTCGAGGTAGGCGTGTACGGTGGGAGGCCTATATAAGCAGAGCTCGTTTAGTGAACCGTCAGATCGCCTGGAG |
| miRNA FF4 | GTGCTCGCTTCGGCAGCACATATACTATGTTGAATGAGGCTTCAGTACTTTACAGAATCGTTGCCTGCACATCTTGGAAACACTTGCTGGGATTACTTCTTCAGGTTAACCCAACAGAAGGCTCGAGTGCTGTTGACAGTGAGCGCCGCTTGAAGTTTTTAATTAAATAGTGAAGCCACAGATGTATTTAATTAAAGACTTCAAGCGGTGCCTACTGCCTCGGAGAATTCAAGGGGCTACTTTAGGAGCAATTATCTTGTTTACTAAAACTGAATACCTTGCTATCTCTTTGATACATTTTTACAAAGCTGAATTAAAATGGTATAAATTAAATCACTTTTTTCAATTG |
| EFS promoter | GGGCAGAGCGCACATCGCCCACAGTCCCCGAGAAGTTGGGGGGAGGGGTCGGCAATTGATCCGGTGCCTAGAGAAGGTGGCGCGGGGTAAACTGGGAAAGTGATGTCGTGTACTGGCTCCGCCTTTTTCCCGAGGGTGGGGGAGAACCGTATATAAGTGCAGTAGTCGCCGTGAACGTTCTTTTTCGCAACGGGTTTGCCGCCAGAACACA |
| 4xNFAT promoter | GGAGGAAAAACTGTTTCATACAGAAGGCGTGGAGGAAAAACTGTTTCATACAGAAGGCGTGGAGGAAAAACTGTTTCATACAGAAGGCGTGGAGGAAAAACTGTTTCATACAGAAGGCGT |
| GD2 CAR | ATGGAGTTGGGGCTGAGCTGGGTTTTCCTTGTTGCTATTTTAGAAGGTGTCCAGTGTGAAGTTCAGCTGCTGCAGAGCGGACCCGAACTGGAAAAACCTGGCGCCTCCGTGATGATCAGCTGCAAGGCCTCTGGCAGCTCCTTCACCGGCTACAACATGAACTGGGTCCGACAGAACATCGGCAAGAGCCTGGAATGGATCGGCGCCATCGATCCTTACTACGGCGGCACCAGCTACAACCAGAAGTTCAAGGGCAGAGCCACACTGACCGTGGACAAGAGCAGCAGCACAGCCTACATGCATCTGAAGTCCCTGACCAGCGAGGACAGCGCCGTGTACTACTGTGTGTCCGGCATGAAGTACTGGGGCCAGGGCACAAGCGTGACAGTCTCTTCTGGCGGCGGTGGATCTGGCGGAGGCGGAAGTGGTGGCGGCGGATCTGATGTGGTCATGACACAGACCCCTCTGAGCCTGCCTGTGTCTCTGGGAGATCAGGCCAGCATCAGCTGTAGAAGCAGCCAGAGCCTGGTGCACAGAAACGGCAACACCTACCTGCACTGGTATCTGCAGAAGCCCGGCCAGTCTCCTAAGCTGCTGATCCACAAGGTGTCCAACAGATTCAGCGGCGTGCCCGACAGATTCTCTGGCTCTGGAAGCGGCACCGACTTCACCCTGAAGATTAGCAGAGTGGAAGCCGAGGACCTGGGCGTGTACTTCTGTAGCCAGAGCACACACGTGCCACCTCTGACATTTGGCGCTGGCACCAAGCTGGAACTGGAGCCCAAATCTTGTGACAAAACTCACACATGCCCACCGTGCCCAGCACCTGAACTCCTGGGGGGACCGTCAGTCTTCCTCTTCCCCCCAAAACCCAAGGACACCCTCATGATCTCCCGGACCCCTGAGGTCACATGCGTGGTGGTGGACGTGAGCCACGAAGACCCTGAGGTCAAGTTCAACTGGTACGTGGACGGCGTGGAGGTGCATAATGCCAAGACAAAGCCGCGGGAGGAGCAGTACAACAGCACGTACCGTGTGGTCAGCGTCCTCACCGTCCTGCACCAGGACTGGCTGAATGGCAAGGAGTACAAGTGCAAGGTCTCCAACAAAGCCCTCCCAGCCCCCATCGAGAAAACCATCTCCAAAGCCAAAGGGCAGCCCCGAGAACCACAGGTGTACACCCTGCCCCCATCCCGGGATGAGCTGACCAAGAACCAGGTCAGCCTGACCTGCCTGGTCAAAGGCTTCTATCCCAGCGACATCGCCGTGGAGTGGGAGAGCAATGGGCAGCCGGAGAACAACTACAAGACCACGCCTCCCGTGCTGGACTCCGACGGCTCCTTCTTCCTCTACAGCAAGCTCACCGTGGACAAGAGCAGGTGGCAGCAGGGGAACGTCTTCTCATGCTCCGTGATGCATGAGGCTCTGCACAACCACTACACACAGAAGAGCCTCTCCCTGTCTCCGGGTAAATTTTGGGTGCTGGTGGTGGTTGGTGGAGTCCTGGCTTGCTATAGCTTGCTAGTAACAGTGGCCTTTATTATTTTCTGGGTGAGGAGTAAGAGGAGCAGGCTCCTGCACAGTGACTACATGAACATGACTCCCCGCCGCCCCGGGCCCACCCGCAAGCATTACCAGCCCTATGCCCCACCACGCGACTTCGCAGCCTATCGCTCCCGCGTCAAGTTCTCACGGTCCGCCGACGCCCCCGCATATCAACAGGGCCAGAATCAGCTCTACAACGAGCTGAACCTGGGAAGGAGAGAGGAGTACGACGTGCTGGACAAGCGACGCGGACGCGACCCGGAGATGGGGGGGAAACCACGGCGGAAAAACCCTCAGGAAGGACTGTACAACGAACTCCAGAAAGACAAGATGGCGGAAGCCTACTCAGAAATCGGGATGAAGGGAGAGCGGAGGAGGGGAAAGGGTCACGACGGGCTGTACCAGGGACTGAGCACCGCCACTAAGGATACCTACGATGCCTTGCATATGCAAGCACTCCCACCCCGG |
| 4 x FF4 binding sites | CCGCTTGAAGTCTTTAATTAAACCGCTTGAAGTCTTTAATTAAACCGCTTGAAGTCTTTAATTAAACCGCTTGAAGTCTTTAATTAAA |
| pEx1 plasmid backbone | CAAAGGATAGAGATAAAAGACACCAAGGAAGCTTTAGACAAGATAGAGGAAGAGCAAAACAAAAGTAAGACCACCGCACAGCAAGCGGCCGCTGATCTTCAGACCTGGAGGAGGAGATATGAGGGACAATTGGAGAAGTGAATTATATAAATATAAAGTAGTAAAAATTGAACCATTAGGAGTAGCACCCACCAAGGCAAAGAGAAGAGTGGTGCAGAGAGAAAAAAGAGCAGTGGGAATAGGAGCTTTGTTCCTTGGGTTCTTGGGAGCAGCAGGAAGCACTATGGGCGCAGCGTCAATGACGCTGACGGTACAGGCCAGACAATTATTGTCTGGTATAGTGCAGCAGCAGAACAATTTGCTGAGGGCTATTGAGGCGCAACAGCATCTGTTGCAACTCACAGTCTGGGGCATCAAGCAGCTCCAGGCAAGAATCCTGGCTGTGGAAAGATACCTAAAGGATCAACAGCTCCTGGGGATTTGGGGTTGCTCTGGAAAACTCATTTGCACCACTGCTGTGCCTTGGAATGCTAGTTGGAGTAATAAATCTCTGGAACAGATTTGGAATCACACGACCTGGATGGAGTGGGACAGAGAAATTAACAATTACACAAGCTTAATACACTCCTTAATTGAAGAATCGCAAAACCAGCAAGAAAAGAATGAACAAGAATTATTGGAATTAGATAAATGGGCAAGTTTGTGGAATTGGTTTAACATAACAAATTGGCTGTGGTATATAAAATTATTCATAATGATAGTAGGAGGCTTGGTAGGTTTAAGAATAGTTTTTGCTGTACTTTCTATAGTGAATAGAGTTAGGCAGGGATATTCACCATTATCGTTTCAGACCCACCTCCCAACCCCGAGGGGACCCGACAGGCCCGAAGGAATAGAAGAAGAAGGTGGAGAGAGAGACAGAGACAGATCCATTCGATTAGTGAACGGATCGGCACTGCGTGCGCCAATTCTGCAGACAAATGGCAGTATTCATCCACAATTTTAAAAGAAAAGGGGGGATTGGGGGGTACAGTGCAGGGGAAAGAATAGTAGACATAATAGCAACAGACATACAAACTAAAGAATTACAAAAACAAATTACAAAAATTCAAAATTTTCGGGTTTATTACAGGGACAGCAGAGATCCAGTTTGGTTAATTAAGGATCCGTTTTCTAGAGTCGAGGTAGGCGTGTACGGTGGGAGGCCTATATAAGCAGAGCTCGTTTAGTGAACCGTCAGATCGCCTGGAGGTGCTCGCTTCGGCAGCACATATACTATGTTGAATGAGGCTTCAGTACTTTACAGAATCGTTGCCTGCACATCTTGGAAACACTTGCTGGGATTACTTCTTCAGGTTAACCCAACAGAAGGCTCGAGTGCTGTTGACAGTGAGCGCCGCTTGAAGTTTTTAATTAAATAGTGAAGCCACAGATGTATTTAATTAAAGACTTCAAGCGGTGCCTACTGCCTCGGAGAATTCAAGGGGCTACTTTAGGAGCAATTATCTTGTTTACTAAAACTGAATACCTTGCTATCTCTTTGATACATTTTTACAAAGCTGAATTAAAATGGTATAAATTAAATCACTTTTTTCAATTGCCCTGATTCTGTGGGCAGAGCGCACATCGCCCACAGTCCCCGAGAAGTTGGGGGGAGGGGTCGGCAATTGATCCGGTGCCTAGAGAAGGTGGCGCGGGGTAAACTGGGAAAGTGATGTCGTGTACTGGCTCCGCCTTTTTCCCGAGGGTGGGGGAGAACCGTATATAAGTGCAGTAGTCGCCGTGAACGTTCTTTTTCGCAACGGGTTTGCCGCCAGAACACAGCCACCATGGAGTTGGGGCTGAGCTGGGTTTTCCTTGTTGCTATTTTAGAAGGTGTCCAGTGTGAAGTTCAGCTGCTGCAGAGCGGACCCGAACTGGAAAAACCTGGCGCCTCCGTGATGATCAGCTGCAAGGCCTCTGGCAGCTCCTTCACCGGCTACAACATGAACTGGGTCCGACAGAACATCGGCAAGAGCCTGGAATGGATCGGCGCCATCGATCCTTACTACGGCGGCACCAGCTACAACCAGAAGTTCAAGGGCAGAGCCACACTGACCGTGGACAAGAGCAGCAGCACAGCCTACATGCATCTGAAGTCCCTGACCAGCGAGGACAGCGCCGTGTACTACTGTGTGTCCGGCATGAAGTACTGGGGCCAGGGCACAAGCGTGACAGTCTCTTCTGGCGGCGGTGGATCTGGCGGAGGCGGAAGTGGTGGCGGCGGATCTGATGTGGTCATGACACAGACCCCTCTGAGCCTGCCTGTGTCTCTGGGAGATCAGGCCAGCATCAGCTGTAGAAGCAGCCAGAGCCTGGTGCACAGAAACGGCAACACCTACCTGCACTGGTATCTGCAGAAGCCCGGCCAGTCTCCTAAGCTGCTGATCCACAAGGTGTCCAACAGATTCAGCGGCGTGCCCGACAGATTCTCTGGCTCTGGAAGCGGCACCGACTTCACCCTGAAGATTAGCAGAGTGGAAGCCGAGGACCTGGGCGTGTACTTCTGTAGCCAGAGCACACACGTGCCACCTCTGACATTTGGCGCTGGCACCAAGCTGGAACTGGAGCCCAAATCTTGTGACAAAACTCACACATGCCCACCGTGCCCAGCACCTGAACTCCTGGGGGGACCGTCAGTCTTCCTCTTCCCCCCAAAACCCAAGGACACCCTCATGATCTCCCGGACCCCTGAGGTCACATGCGTGGTGGTGGACGTGAGCCACGAAGACCCTGAGGTCAAGTTCAACTGGTACGTGGACGGCGTGGAGGTGCATAATGCCAAGACAAAGCCGCGGGAGGAGCAGTACAACAGCACGTACCGTGTGGTCAGCGTCCTCACCGTCCTGCACCAGGACTGGCTGAATGGCAAGGAGTACAAGTGCAAGGTCTCCAACAAAGCCCTCCCAGCCCCCATCGAGAAAACCATCTCCAAAGCCAAAGGGCAGCCCCGAGAACCACAGGTGTACACCCTGCCCCCATCCCGGGATGAGCTGACCAAGAACCAGGTCAGCCTGACCTGCCTGGTCAAAGGCTTCTATCCCAGCGACATCGCCGTGGAGTGGGAGAGCAATGGGCAGCCGGAGAACAACTACAAGACCACGCCTCCCGTGCTGGACTCCGACGGCTCCTTCTTCCTCTACAGCAAGCTCACCGTGGACAAGAGCAGGTGGCAGCAGGGGAACGTCTTCTCATGCTCCGTGATGCATGAGGCTCTGCACAACCACTACACACAGAAGAGCCTCTCCCTGTCTCCGGGTAAATTTTGGGTGCTGGTGGTGGTTGGTGGAGTCCTGGCTTGCTATAGCTTGCTAGTAACAGTGGCCTTTATTATTTTCTGGGTGAGGAGTAAGAGGAGCAGGCTCCTGCACAGTGACTACATGAACATGACTCCCCGCCGCCCCGGGCCCACCCGCAAGCATTACCAGCCCTATGCCCCACCACGCGACTTCGCAGCCTATCGCTCCCGCGTCAAGTTCTCACGGTCCGCCGACGCCCCCGCATATCAACAGGGCCAGAATCAGCTCTACAACGAGCTGAACCTGGGAAGGAGAGAGGAGTACGACGTGCTGGACAAGCGACGCGGACGCGACCCGGAGATGGGGGGGAAACCACGGCGGAAAAACCCTCAGGAAGGACTGTACAACGAACTCCAGAAAGACAAGATGGCGGAAGCCTACTCAGAAATCGGGATGAAGGGAGAGCGGAGGAGGGGAAAGGGTCACGACGGGCTGTACCAGGGACTGAGCACCGCCACTAAGGATACCTACGATGCCTTGCATATGCAAGCACTCCCACCCCGGGAGGGCCGCGGCAGCCTGCTGACCTGCGGCGACGTGGAGGAAAACCCCGGCCCCACCGGTATGAGCGAGCTGATTAAGGAGAACATGCACATGAAGCTGTACATGGAGGGCACCGTGGACAACCATCACTTCAAGTGCACATCCGAGGGCGAAGGCAAGCCCTACGAGGGCACCCAGACCATGAGAATCAAGGTGGTCGAGGGCGGCCCTCTCCCCTTCGCCTTCGACATCCTGGCTACTAGCTTCCTCTACGGCAGCAAGACCTTCATCAACCACACCCAGGGCATCCCCGACTTCTTCAAGCAGTCCTTCCCTGAGGGCTTCACATGGGAGAGAGTCACCACATACGAAGACGGGGGCGTGCTGACCGCTACCCAGGACACCAGCCTCCAGGACGGCTGCCTCATCTACAACGTCAAGATCAGAGGGGTGAACTTCACATCCAACGGCCCTGTGATGCAGAAGAAAACACTCGGCTGGGAGGCCTTCACCGAGACGCTGTACCCCGCTGACGGCGGCCTGGAAGGCAGAAACGACATGGCCCTGAAGCTCGTGGGCGGGAGCCATCTGATCGCAAACATCAAGACCACATATAGATCCAAGAAACCCGCTAAGAACCTCAAGATGCCTGGCGTCTACTATGTGGACTACAGACTGGAAAGAATCAAGGAGGCCAACAACGAGACCTACGTCGAGCAGCACGAGGTGGCAGTGGCCAGATACTGCGACCTCCCTAGCAAACTGGGGCACTAAGAGCGGCCGCAAAGCTTCGAATTCCGCTTGAAGTCTTTAATTAAACCGCTTGAAGTCTTTAATTAAACCGCTTGAAGTCTTTAATTAAACCGCTTGAAGTCTTTAATTAAAGTACCGCGGGCCCGGCTAGATAACTGATCTACCCAGCTTTCTTGTACAAAGTGGTGAATTCCGGCAATTCGATATCAAGCTTATCGATAATCAACCTCTGGATTACAAAATTTGTGAAAGATTGACTGGTATTCTTAACTATGTTGCTCCTTTTACGCTATGTGGATACGCTGCTTTAATGCCTTTGTATCATGCTATTGCTTCCCGTATGGCTTTCATTTTCTCCTCCTTGTATAAATCCTGGTTGCTGTCTCTTTATGAGGAGTTGTGGCCCGTTGTCAGGCAACGTGGCGTGGTGTGCACTGTGTTTGCTGACGCAACCCCCACTGGTTGGGGCATTGCCACCACCTGTCAGCTCCTTTCCGGGACTTTCGCTTTCCCCCTCCCTATTGCCACGGCGGAACTCATCGCCGCCTGCCTTGCCCGCTGCTGGACAGGGGCTCGGCTGTTGGGCACTGACAATTCCGTGGTGTTGTCGGGGAAATCATCGTCCTTTCCTTGGCTGCTCGCCTGTGTTGCCACCTGGATTCTGCGCGGGACGTCCTTCTGCTACGTCCCTTCGGCCCTCAATCCAGCGGACCTTCCTTCCCGCGGCCTGCTGCCGGCTCTGCGGCCTCTTCCGCGTCTTCGCCTTCGCCCTCAGACGAGTCGGATCTCCCTTTGGGCCGCCTCCCCGCATCGATACCGTCGACCTCGAGTCCTAGAAAAACATGGAGCAATCACAAGTAGCAATACAGCAGCTACCAATGCTGATTGTGCCTGGCTAGAAGCACAAGAGGAGGAGGAGGTGGGTTTTCCAGTCACACCTCAGGTACCTTTAAGACCAATGACTTACAAGGCAGCTGTAGATCTTAGCCACTTTTTAAAAGAAAAGGGGGGACTGGAAGGGCTAATTCACTCCCAACGAAGACAAGATATCCTTGATCTGTGGATCTACCACACACAAGGCTACTTCCCTGATTGGCAGAACTACACACCAGGGCCAGGGATCAGATATCCACTGACCTTTGGATGGTGCTACAAGCTAGTACCAGTTGAGCAAGAGAAGGTAGAAGAAGCCAATGAAGGAGAGAACACCCGCTTGTTACACCCTGTGAGCCTGCATGGGATGGATGACCCGGAGAGAGAAGTATTAGAGTGGAGGTTTGACAGCCGCCTAGCATTTCATCACATGGCCCGAGAGCTGCATCCGGACTGTACTGGGTGTCTCTGGTTAGACCAGATCTGAGCCTGGGAGCTCTCTGGCTAACTAGGGAACCCACTGCTTAAGCCTCAATAAAGCTTGCCTTGAGTGCTTCAAGTAGTGTGTGCCCGTCTGTTGTGTGACTCTGGTAACTAGAGATCCCTCAGACCCTTTTAGTCAGTGTGGAAAATCTCTAGCAGGGCCCGTTTAAACCCGCTGATCAGCCTCGACTGTGCCTTCTAGTTGCCAGCCATCTGTTGTTTGCCCCTCCCCCGTGCCTTCCTTGACCCTGGAAGGTGCCACTCCCACTGTCCTTTCCTAATAAAATGAGGAAATTGCATCGCATTGTCTGAGTAGGTGTCATTCTATTCTGGGGGGTGGGGTGGGGCAGGACAGCAAGGGGGAGGATTGGGAAGACAATAGCAGGCATGCTGGGGATGCGGTGGGCTCTATGGCTTCTGAGGCGGAAAGAACCAGCTGGGGCTCTAGGGGGTATCCCCACGCGCCCTGTAGCGGCGCATTAAGCGCGGCGGGTGTGGTGGTTACGCGCAGCGTGACCGCTACACTTGCCAGCGCCCTAGCGCCCGCTCCTTTCGCTTTCTTCCCTTCCTTTCTCGCCACGTTCGCCGGCTTTCCCCGTCAAGCTCTAAATCGGGGGCTCCCTTTAGGGTTCCGATTTAGTGCTTTACGGCACCTCGACCCCAAAAAACTTGATTAGGGTGATGGTTCACGTAGTGGGCCATCGCCCTGATAGACGGTTTTTCGCCCTTTGACGTTGGAGTCCACGTTCTTTAATAGTGGACTCTTGTTCCAAACTGGAACAACACTCAACCCTATCTCGGTCTATTCTTTTGATTTATAAGGGATTTTGCCGATTTCGGCCTATTGGTTAAAAAATGAGCTGATTTAACAAAAATTTAACGCGAATTAATTCTGTGGAATGTGTGTCAGTTAGGGTGTGGAAAGTCCCCAGGCTCCCCAGCAGGCAGAAGTATGCAAAGCATGCATCTCAATTAGTCAGCAACCAGGTGTGGAAAGTCCCCAGGCTCCCCAGCAGGCAGAAGTATGCAAAGCATGCATCTCAATTAGTCAGCAACCATAGTCCCGCCCCTAACTCCGCCCATCCCGCCCCTAACTCCGCCCAGTTCCGCCCATTCTCCGCCCCATGGCTGACTAATTTTTTTTATTTATGCAGAGGCCGAGGCCGCCTCTGCCTCTGAGCTATTCCAGAAGTAGTGAGGAGGCTTTTTTGGAGGCCTAGGCTTTTGCAAAAAGCTCCCGGGAGCTTGTATATCCATTTTCGGATCTGATCAGCACGTGTTGACAATTAATCATCGGCATAGTATATCGGCATAGTATAATACGACAAGGTGAGGAACTAAACCATGGCCAAGTTGACCAGTGCCGTTCCGGTGCTCACCGCGCGCGACGTCGCCGGAGCGGTCGAGTTCTGGACCGACCGGCTCGGGTTCTCCCGGGACTTCGTGGAGGACGACTTCGCCGGTGTGGTCCGGGACGACGTGACCCTGTTCATCAGCGCGGTCCAGGACCAGGTGGTGCCGGACAACACCCTGGCCTGGGTGTGGGTGCGCGGCCTGGACGAGCTGTACGCCGAGTGGTCGGAGGTCGTGTCCACGAACTTCCGGGACGCCTCCGGGCCGGCCATGACCGAGATCGGCGAGCAGCCGTGGGGGCGGGAGTTCGCCCTGCGCGACCCGGCCGGCAACTGCGTGCACTTCGTGGCCGAGGAGCAGGACTGACACGTGCTACGAGATTTCGATTCCACCGCCGCCTTCTATGAAAGGTTGGGCTTCGGAATCGTTTTCCGGGACGCCGGCTGGATGATCCTCCAGCGCGGGGATCTCATGCTGGAGTTCTTCGCCCACCCCAACTTGTTTATTGCAGCTTATAATGGTTACAAATAAAGCAATAGCATCACAAATTTCACAAATAAAGCATTTTTTTCACTGCATTCTAGTTGTGGTTTGTCCAAACTCATCAATGTATCTTATCATGTCTGTATACCGTCGACCTCTAGCTAGAGCTTGGCGTAATCATGGTCATAGCTGTTTCCTGTGTGAAATTGTTATCCGCTCACAATTCCACACAACATACGAGCCGGAAGCATAAAGTGTAAAGCCTGGGGTGCCTAATGAGTGAGCTAACTCACATTAATTGCGTTGCGCTCACTGCCCGCTTTCCAGTCGGGAAACCTGTCGTGCCAGCTGCATTAATGAATCGGCCAACGCGCGGGGAGAGGCGGTTTGCGTATTGGGCGCTCTTCCGCTTCCTCGCTCACTGACTCGCTGCGCTCGGTCGTTCGGCTGCGGCGAGCGGTATCAGCTCACTCAAAGGCGGTAATACGGTTATCCACAGAATCAGGGGATAACGCAGGAAAGAACATGTGAGCAAAAGGCCAGCAAAAGGCCAGGAACCGTAAAAAGGCCGCGTTGCTGGCGTTTTTCCATAGGCTCCGCCCCCCTGACGAGCATCACAAAAATCGACGCTCAAGTCAGAGGTGGCGAAACCCGACAGGACTATAAAGATACCAGGCGTTTCCCCCTGGAAGCTCCCTCGTGCGCTCTCCTGTTCCGACCCTGCCGCTTACCGGATACCTGTCCGCCTTTCTCCCTTCGGGAAGCGTGGCGCTTTCTCATAGCTCACGCTGTAGGTATCTCAGTTCGGTGTAGGTCGTTCGCTCCAAGCTGGGCTGTGTGCACGAACCCCCCGTTCAGCCCGACCGCTGCGCCTTATCCGGTAACTATCGTCTTGAGTCCAACCCGGTAAGACACGACTTATCGCCACTGGCAGCAGCCACTGGTAACAGGATTAGCAGAGCGAGGTATGTAGGCGGTGCTACAGAGTTCTTGAAGTGGTGGCCTAACTACGGCTACACTAGAAGAACAGTATTTGGTATCTGCGCTCTGCTGAAGCCAGTTACCTTCGGAAAAAGAGTTGGTAGCTCTTGATCCGGCAAACAAACCACCGCTGGTAGCGGTGGTTTTTTTGTTTGCAAGCAGCAGATTACGCGCAGAAAAAAAGGATCTCAAGAAGATCCTTTGATCTTTTCTACGGGGTCTGACGCTCAGTGGAACGAAAACTCACGTTAAGGGATTTTGGTCATGACCAGCCAGGACAGAAATGCCTCGACTTCGCTGCTACCCAAGGTTGCCGGGTGACGCACACCGTGGAAACGGATGAAGGCACGAACCCAGTGGACATAAGCCTGTTCGGTTCGTAAGCTGTAATGCAAGTAGCGTATGCGCTCACGCAACTGGTCCAGAACCTTGACCGAACGCAGCGGTGGTAACGGCGCAGTGGCGGTTTTCATGGCTTGTTATGACTGTTTTTTTGGGGTACAGTCTATGCCTCGGGCATCCAAGCAGCAAGCGCGTTACGCCGTGGGTCGATGTTTGATGTTATGGAGCAGCAACGATGTTACGCAGCAGGGCAGTCGCCCTAAAACAAAGTTAAACATTATGAGGGAAGCGGTGATCGCCGAAGTATCGACTCAACTATCAGAGGTAGTTGGCGTCATCGAGCGCCATCTCGAACCGACGTTGCTGGCCGTACATTTGTACGGCTCCGCAGTGGATGGCGGCCTGAAGCCACACAGTGATATTGATTTGCTGGTTACGGTGACCGTAAGGCTTGATGAAACAACGCGGCGAGCTTTGATCAACGACCTTTTGGAAACTTCGGCTTCCCCTGGAGAGAGCGAGATTCTCCGCGCTGTAGAAGTCACCATTGTTGTGCACGACGACATCATTCCGTGGCGTTATCCAGCTAAGCGCGAACTGCAATTTGGAGAATGGCAGCGCAATGACATTCTTGCAGGTATCTTCGAGCCAGCCACGATCGACATTGATCTGGCTATCTTGCTGACAAAAGCAAGAGAACATAGCGTTGCCTTGGTAGGTCCAGCGGCGGAGGAACTCTTTGATCCGGTTCCTGAACAGGATCTATTTGAGGCGCTAAATGAAACCTTAACGCTATGGAACTCGCCGCCCGACTGGGCTGGCGATGAGCGAAATGTAGTGCTTACGTTGTCCCGCATTTGGTACAGCGCAGTAACCGGCAAAATCGCGCCGAAGGATGTCGCTGCCGACTGGGCAATGGAGCGCCTGCCGGCCCAGTATCAGCCCGTCATACTTGAAGCTAGACAGGCTTATCTTGGACAAGAAGAAGATCGCTTGGCCTCGCGCGCAGATCAGTTGGAAGAATTTGTCCACTACGTGAAAGGCGAGATCACCAAGGTAGTCGGCAAATAATCATGAGCGGATACATATTTGAATGTATTTAGAAAAATAAACAAATAGGGGTTCCGCGCACATTTCCCCGAAAAGTGCCACCTGACGTCGACGGATCGGGAGATCTCCCGATCCCCTATGGTGCACTCTCAGTACAATCTGCTCTGATGCCGCATAGTTAAGCCAGTATCTGCTCCCTGCTTGTGTGTTGGAGGTCGCTGAGTAGTGCGCGAGCAAAATTTAAGCTACAACAAGGCAAGGCTTGACCGACAATTGCATGAAGAATCTGCTTAGGGTTAGGCGTTTTGCGCTGCTTCGCGATGTACGGGCCAGATATACGCGTTGACATTGATTATTGACTAGTTATTAATAGTAATCAATTACGGGGTCATTAGTTCATAGCCCATATATGGAGTTCCGCGTTACATAACTTACGGTAAATGGCCCGCCTGGCTGACCGCCCAACGACCCCCGCCCATTGACGTCAATAATGACGTATGTTCCCATAGTAACGCCAATAGGGACTTTCCATTGACGTCAATGGGTGGAGTATTTACGGTAAACTGCCCACTTGGCAGTACATCAAGTGTATCATATGCCAAGTACGCCCCCTATTGACGTCAATGACGGTAAATGGCCCGCCTGGCATTATGCCCAGTACATGACCTTATGGGACTTTCCTACTTGGCAGTACATCTACGTATTAGTCATCGCTATTACCATGGTGATGCGGTTTTGGCAGTACATCAATGGGCGTGGATAGCGGTTTGACTCACGGGGATTTCCAAGTCTCCACCCCATTGACGTCAATGGGAGTTTGTTTTGGCACCAAAATCAACGGGACTTTCCAAAATGTCGTAACAACTCCGCCCCATTGACGCAAATGGGCGGTAGGCGTGTACGGTGGGAGGTCTATATAAGCAGCGCGTTTTGCCTGTACTGGGTGTCTCTGGTTAGACCAGATCTGAGCCTGGGAGCTCTCTGGCTAACTAGGGAACCCACTGCTTAAGCCTCAATAAAGCTTGCCTTGAGTGCTTCAAGTAGTGTGTGCCCGTCTGTTGTGTGACTCTGGTAACTAGAGATCCCTCAGACCCTTTTAGTCAGTGTGGAAAATCTCTAGCAGTGGCGCCCGAACAGGGACTTGAAAGCGAAAGGGAAACCAGAGGAGCTCTCTCGACGCAGGACTCGGCTTGCTGAAGCGCGCACGGCAAGAGGCGAGGGGCGGCGACTGGTGAGTACGCCAAAAATTTTGACTAGCGGAGGCTAGAAGGAGAGAGATGGGTGCGAGAGCGTCAGTATTAAGCGGGGGAGAATTAGATCGCGATGGGAAAAAATTCGGTTAAGGCCAGGGGGAAAGAAAAAATATAAATTAAAACATATAGTATGGGCAAGCAGGGAGCTAGAACGATTCGCAGTTAATCCTGGCCTGTTAGAAACATCAGAAGGCTGTAGACAAATACTGGGACAGCTACAACCATCCCTTCAGACAGGATCAGAAGAACTTAGATCATTATATAATACAGTAGCAACCCTCTATTGTGTGCAT |
